# Research on Hot Deformation Rheological Stress of Al-Mg-Si-Mn-Sc Aluminium Alloy

**DOI:** 10.3390/ma17133159

**Published:** 2024-06-27

**Authors:** Wei Sun, Yu Zhang, Fang Yu, Lingfei Yang, Dongfu Song, Guozhong He, Weiping Tong, Xiangjie Wang

**Affiliations:** 1 Key Lab of Electromagnetic Processing of Materials, Ministry of Education, Northeastern University, Shenyang 110819, China; sunwei2020456@163.com (W.S.); yufang1989@163.com (F.Y.); 2010206@stu.neu.edu.cn (L.Y.); wptong@mail.neu.edu.cn (W.T.); 2 Liaoning Zhongwang Group Research Institute, Liaoyang 111003, China; zhangyu201507@163.com; 3 Guangdong Haomei Technology Research Institute Co., Ltd., Qingyuan 511599, China; songyuren1015@163.com; 4 Shihezi Zhonghe New Materials Co., Ltd., Shihezi 832000, China

**Keywords:** Al-Mg-Si-Mn alloy, rare earth Sc, rheological stress, thermal deformation activation energy, hot working diagram

## Abstract

The hot compression simulation testing machine was utilized to conduct compression experiments on an Al-Mg-Si-Mn alloy containing the rare earth element Sc at a deformation temperature ranging from 450 to 550 °C and a strain rate of 0.01 to 10 s^−1^. The study focused on the hot deformation behavior of the aluminum alloy, resulting in the determination of the optimal range of deformation process parameters for the alloy. The relationship between material flow stress, deformation temperature, and strain rate was described using the Arrhenius relationship containing thermal activation energy based on the stress-strain curve of hot compression deformation of aluminum alloy. This led to calculations for structural factor A, stress index n, and stress level parameters as well as thermal deformation activation energy to establish a constitutive Formula for hot deformation rheological stress of aluminum alloy and calculate the power dissipation factor η. Through this process, an optimized range for the optimal deformation process parameter for aluminum alloy was determined (deformation temperature: 490~510 °C; strain rate: 0.05 s^−1^) and verified in combination with mechanical properties and microstructure through hot extrusion deformation trial production.

## 1. Introduction

In the current context of the rapid development of electric vehicles, there is a growing focus on their driving range. Aluminum alloy plays a crucial role as a lightweight material in automobile design due to its advantageous properties such as low density, high strength, ease of processing, and recyclability [1]. For instance, Al-Mg-Si alloy, exemplified by 6005A alloy with a final (T6) tensile strength reaching 300 MPa, is commonly used in automotive structures. However, challenges persist in the application of aluminum alloys including significant reduction in mechanical properties under prolonged exposure to high temperatures and alternating loads. These issues can compromise vehicle safety and limit the potential applications of aluminum alloys [2,3,4].

Alloying is currently the main means of improving alloy properties [5], and Sc is a highly chemically active transition element. Due to its similar outer electronic structure to rare earth-containing alloys, it is also listed as a rare earth element [6]. Sc has a prominent effect on aluminum alloys, mainly due to modification [7], purification [8], and microalloying [9]. Iwamura S. et al. [10] studied the effect of Sc on Al-Mg alloy and found that the alloy’s yield strength and tensile strength increased by about 90 MPa and 120 MPa, respectively. The elongation after fracture remained at a high level, above 15%. Tian S.K. et al. [11] investigated the effect of Sc (Zr) on 7136 alloy and found that Sc element can promote nonspontaneous nucleation of the alloy, with a Sc element addition of 0.2 wt.% At this time, the strength of the alloy increased by 36.63 MPa. At present, research on Sc element in aluminum alloys mainly focuses on Al-Mg, Al-Zn-Mg (Cu) based aluminum alloys [12,13,14], Al-Mg-Si alloy is a commonly used automotive structural material. Compared with Al-Mg and Al-Zn-Mg alloys, Al-Mg-Si alloy has higher extrusion properties and collision resistance, but the general strength of the series of alloys is low, which limits the application of the material. while there is relatively little research on Sc in Al-Mg-Si-based deformed aluminum alloys, and there is no research on its hot deformation flow stress during extrusion trial production. In previous studies, there were relatively few experiments on the Sc addition of Al-Mg-Si alloy and almost no studies on the thermal deformation flow stress of this kind of alloy. Therefore, there is no good reference basis in terms of the research gap.

To improve the application range of Sc in deformed aluminum alloys and assist in the lightweight technology of automotive structures. This article systematically studied the flow stress of Al-Mg-Si alloy used for Sc-containing automotive battery pack trays during hot deformation, and drew a hot working diagram based on the flow stress characteristics of the alloy, analyzing the processing window of aluminum alloy.

## 2. Experimental Methods

### 2.1. Material Preparation

Using pure aluminum alloys (99.85 wt.%), magnesium alloys (99.95 wt.%), industrial silicon (98.50 wt.%), Al-10Mn, Al-60Cu, Al-20Cr, and Al-2Sc master alloys as raw materials. Heat pure aluminum alloy in the melting furnace until it is completely melted, add industrial silicon, Al-Cu, Al-Mn, and Al-Cr master alloys, heat, and stir until the master alloy is completely melted, and add pure magnesium ingots and Al-Sc master alloys. Refine using pure argon gas (99.99 wt.%), add refining agent Al-Ti-5B wire to the melting point during drainage, pass through the degassing box and filter box, and then enter the casting plate crystallizer for casting. The chemical composition of the processed ingot was analyzed according to GB/T 7999-2007 [15]. Optical emission spectrometric analysis method of aluminum and aluminum alloys; the alloy composition was measured by Prodigy7 photoelectric direct reading emission spectrometry (Teledyne Leeman Labs., Hudson, NH, USA). The composition of aluminum alloy is shown in Table 1. The ingots are subjected to phased homogenization treatment under industrial production conditions, with the furnace temperature raised to 350 °C for 2 h and then raised again to 560 °C for 8 h.

### 2.2. Test Methods

The hot compression test was used to study the hot deformation rheological behavior of rare earth-containing aluminum alloy, determine the hot working process window of Al-Mg-Si-Mn-Sc alloy, and verify the hot extrusion process of rare earth-containing aluminum alloy profile.

**Figure 1:**
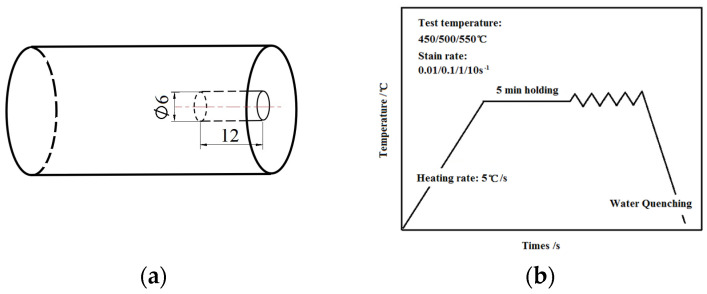
Hot compression test (unit: mm): (**a**) Sample size diagram; (**b**) Test technology.

Samples were taken along the extrusion direction of the ingot billet, and hot compression tests were conducted using the MMS-200 thermal simulation testing machine (Corange Co., Ltd., Shenyang, China). The thermal compression experiments refer to T/CSTM 00244-2021 [16] “Physical Simulation test method for Metal materials Part 1: Axisymmetric thermal compression test” and ASTM F2837-2011(R2018) [17] standard test method for thermal compression properties of gasket materials. To ensure that the specimen is in a unidirectional compression state, copper gaskets with a thickness of 0.1 mm are added at both ends of the specimen to provide isolation and lubrication. The heating and insulation rate is 5 °C/min, and the insulation time is 5 min. The deformation temperatures are 450 °C, 500 °C, and 550 °C, respectively. The strain rates are 0.01 s^−1^, 0.1 s^−1^, 1.0 s^−1^ and 10 s^−1^, respectively. The hot compression test schematic diagram and technology are shown in Figure 1. The compression rate is set to 60%. The test technical parameters of the hot compression sample are shown in Table 2.

The metallographic microstructure was observed using an Imager M2m optical microscope produced by ZEISS company in Aalen, Germany. The microstructure samples were prepared by GB/T 3246.1-2012 [18] “Inspection method for the structure of wrought aluminum and aluminum alloy products-Part 1: Inspection method for microstructure”. The sample undergoes rough grinding (180 # sandpaper), fine grinding (400–1500 # sandpaper), and polishing (particle size ranging from 2.5 to 3.5 μm Diamond polishing paste) After using diamond polishing paste, Keller reagent was used as the etching solution (ratio: 2.5 mL HNO_3_ + 1.5 mL HCL + 1 mL HF + 95 mL H_2_O) to corrode the surface of the sample. Using the sample as the anode, the grain structure is observed by coating it with an applied current through the electrolyte.

The sample was prepared according to the requirements of YS/T 1623-2023 [19] “Inspection of aging precipitates for aluminum alloys-Transmission electron microscopy method”. The sample undergoes rough grinding (2000 # sandpaper) and grinding (sample thickness < 50 μm) After punching (taking a small circular plate with a diameter of 3 mm and electrolytic double spraying until perforation) and double spraying thinning (electrolytic polishing on an electrolytic double spraying instrument with a 25% nitric acid methanol solution as the electrolyte), the microstructure of the sample was observed using a JEOL-F200 transmission electron microscope (JEOL Co., Ltd., Tokyo, Japan) and calibrated by electron diffraction.

## 3. Experimental Results and Analysis

### 3.1. True Stress-Strain Curve

**Figure 2:**
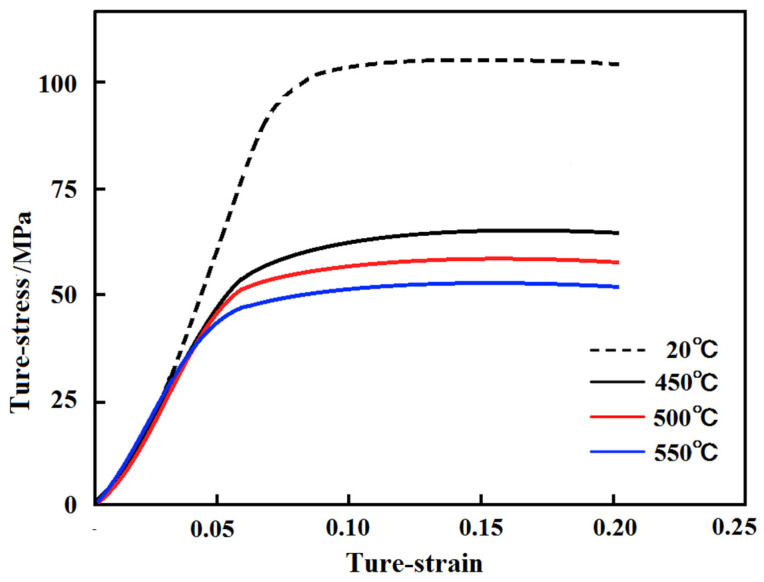
Quasi-static true stress-strain curves of Al-Mg-Si-Mn-Sc alloy at different ambient temperatures.

Figure 2 shows the quasi-static stress-strain curves of Al-Mg-Si-Mn-Sc alloy samples at room temperature 20 °C and high temperatures of 400, 450, and 500 °C. As can be seen from Figure 2, the average yield stress of the room temperature curve is 106 ± 5 MPa, while that of the high-temperature curve is generally low. With the gradual temperature increase, the yield strength drops to 49 ± 5 MPa. The yield strength and ultimate compressive strength at high temperatures are lower than that at room temperature. This may also be due to the thermal softening effect [20], when the deformation temperature increases, the flow stress decreases significantly.

**Figure 3:**
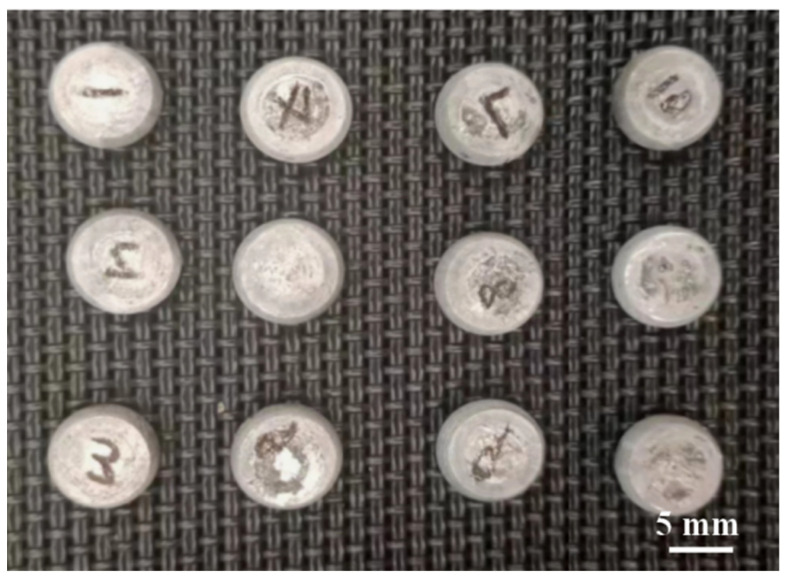
Hot compression specimen of aluminum alloy.

The sample of aluminum alloy after hot compression is shown in Figure 3. All samples did not show significant cracking.

**Figure 4:**
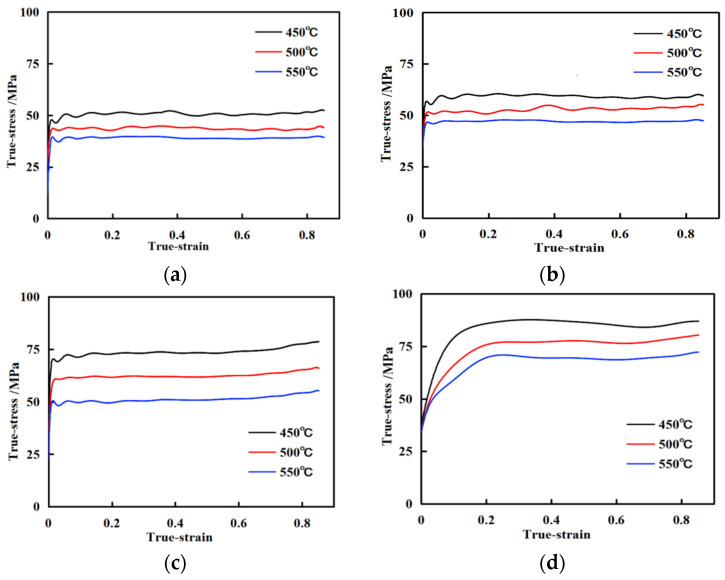
True stress-strain curve of aluminum alloy at different strain rates: (**a**) 0.01 s^−1^; (**b**) 0.1 s^−1^; (**c**) 1.0 s^−1^; (**d**) 10 s^−1^.

The true stress-strain curves of aluminum alloys under different hot compression conditions are shown in Figure 4. As shown in the figure, at the same heating temperature, as the strain rate increases, the stress gradually increases; At the same strain rate, as the deformation temperature increases, the stress gradually decreases, indicating that the alloy is a positive strain rate sensitive material.

In the thermoplastic deformation of metals, there are processes of work hardening and dynamic softening [21]. Metal deformation can cause an increase in dislocations, and the interaction between dislocations leads to alloy hardening. Under deformation conditions, due to the high stacking fault energy and narrow width of extended dislocations in aluminum alloys, cross-slip and climb are prone to occur. Under thermal activation and external pressure, recrystallization nucleation occurs, which can cause material softening [22,23,24]. When the rate of dislocation proliferation cannot continue to meet the requirements of recrystallization nucleation, the softening effect weakens and work hardening becomes dominant again. When the dislocations accumulate again to a certain extent, recrystallization softening occurs again, and this process is cyclic. When the strain rates of aluminum alloy are 0.01 s^−1^ and 0.1 s^−1^, there is cyclic stress, and the two processes of work hardening and dynamic softening compete. The true stress-strain curve shows a wavy characteristic; When the strain rate is 1.0 s^−1^, there is still periodic fluctuation in the early stage of stress. When the strain increases to a certain value, the competition gradually tends to balance, and as the strain increases, the stress also tends to stabilize; When the strain rate is 10 s^−1^; The processes of work hardening and dynamic softening are almost completed simultaneously, and work hardening and dynamic softening reach equilibrium in a short period. The true stress-strain curve is smooth and exhibits steady-state rheological characteristics. At the same time, in the process of hot compression deformation of aluminum alloy, the shape and size of the second phase will change, which will further affect the hot deformation flow stress of the alloy. For example, the possible single crystal Si phase in the alloy will become coarse when the aluminum alloy reaches the recrystallization temperature, which will also affect the stress-strain curve of the alloy [25].

### 3.2. Thermal Deformation Constitutive Formula

During hot compression deformation, the flow stress of as-cast Al-Mg-Si alloy is susceptible to deformation temperature and strain rate. To further understand the relationship between various factors and flow stress, to control the high-temperature plastic deformation behavior of the alloy. The hot deformation of aluminum alloy needs to be carried out in the stable deformation stage and quasi-stable rheological stage, where the material undergoes uniform deformation and the strain is uniformly distributed during the deformation process, mainly divided into work hardening and strain rate hardening stages. Hollomon and Zener proposed the Formula of state for metal flow [26], which can be represented by Formula (1). The relationship between material deformation velocity and deformation temperature is verified, so it is also called temperature-compensated deformation velocity, and many phenomena of plastic machining metals can be explained by this parameter.

(1) σ=σ(Z,ε)

In the formula, σ is the rheological stress, Mepsilon is the true strain; and *Z* is the formula parameter and the deformation rate factor for temperature compensation. It can be represented by Formula (2).

(2) $$Z=\dot{\varepsilon }exp\left[Q/(RT)\right]=A{\left[sinh(\alpha \sigma )\right]}^{n}$$

In the formula, *Q* is the activation energy for thermal deformation, reflecting the difficulty of material thermal deformation, kJ/mol; $\dot{\varepsilon }$ is the strain rate, ^s−1^; *T* is the deformation temperature, K; *R* is the molar gas constant, J/(mol · K); α is a stress level parameter, MPa^−1^, *A* is the structural factor, s^−1^. Sellers and Tegart proposed a hyperbolic sine form Arrhenius correction function based on Formulas (1) and (2) to represent the relationship between rheological stress, strain rate, and temperature. The research results indicate that [27,28,29], Formula (3) can better describe the conventional thermal deformation of metals.

(3) $$\dot{\varepsilon }=A{\left[sinh(\alpha \sigma )\right]}^{n}exp\left[-Q/(RT)\right]$$

Stay Alphaασ Formula (3) can be simplified for different values, i.e., different stress levels. When the stress level is low (ασ the value is generally < 0.8), for the Formula (3) sinh(ασ) after performing a numerical expansion, higher-order terms can be ignored, and the expression is shown in Formula (4) [4].

(4) $$\dot{\varepsilon }=A\sigma^{n}exp\left[-Q/(RT)\right]$$

When the stress level is high (ασ > 1.2), Formula (3) can be ignored ${\left[sinh(\alpha \sigma )\right]}^{n}$ middle exp(ασ) the expression for is shown in Formula (5).

(5) $$\dot{\varepsilon }=A\prime exp(\alpha n\sigma )exp\left[-Q/(RT)\right]$$

Assuming that the alloy satisfies the conventional high-temperature plastic deformation of metals, it is necessary to cope with flow stress σ true strain ε the deformation temperature *T* satisfies Formula (3). Based on the formula of the alloy under high stress and low-stress levels calculate the value in the formula α, *n*. By taking the logarithm of Formulas (4) and (5) respectively, Formulas (6) and (7) can be obtained.

(6) $$ln\dot{\varepsilon }=lnA-Q/(RT)+nln\sigma$$

(7) $$ln\dot{\varepsilon }=lnA\prime -Q/(RT)+\alpha n\sigma$$

**Figure 5:**
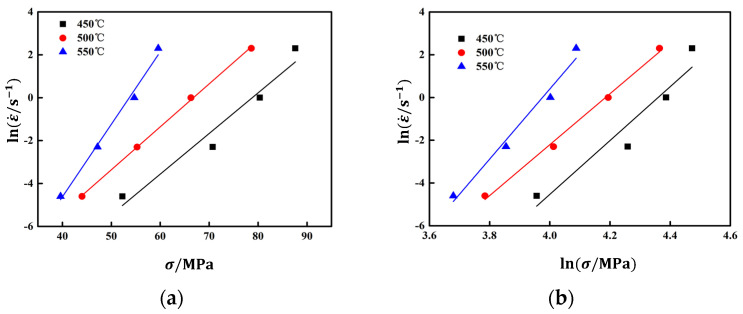
The linear relationship fitting at peak stress: (**a**) $ln(\dot{\varepsilon })-\sigma$ (**b**) $ln(\dot{\varepsilon })-ln(\sigma )$.

According to Formulas (6) and (7), it can be inferred that when the deformation temperature *T* is constant, *n* and αn respectively $ln\dot{\varepsilon }$-lnσ and $ln\dot{\varepsilon }$-σ the slope of. Take the peak stress and strain rate of the alloy under different deformation conditions, and use the univariate linear regression method to obtain the fitting curves at each temperature, $ln\dot{\varepsilon }$-σ, $ln\dot{\varepsilon }$-lnσ the relationship curve is shown in Figure 5.

$ln\dot{\varepsilon }$-σ, $ln\dot{\varepsilon }$-lnσ the linear correlation coefficients of the relationship curve are greater than 0.976 and 0.958, respectively. According to the calculation, *n* for 13.63, αn 0.242, obtain α it is 0.0178 MPa^−1^. Hypothesis Q there is no significant correlation with *T*, taking the logarithm of Formula (5) yields Formula (8), Q can be calculated according to Formula (9).

(8) $$ln\dot{\varepsilon }=lnA-Q/(RT)+nln\left[sinh(\alpha \sigma )\right]$$

(9) $$Q=R{\left\{\frac{\partial ln\dot{\varepsilon }}{\partial \left[sinh(\alpha \sigma )\right]}\right\}}_{T}\left\{\frac{\partial \left[sinh(\alpha \sigma )\right]}{\partial \left(1/T\right)}\right\}$$

**Figure 6:**
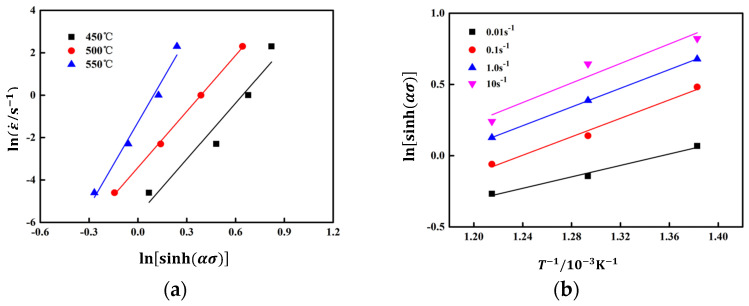
The linear relationship fitting at peak stress: (**a**) $ln\dot{\varepsilon }-ln\left[sinh(\alpha \sigma )\right]$ (**b**) $ln\left[sinh(\alpha \sigma )\right]-1/T$.

Take the peak stress and strain rate of the alloy, as well as the stress level parameters obtained from the previous text α substitute into Formula (8), draw $ln\dot{\varepsilon }$ − $ln\left[sinh(\alpha \sigma )\right]$, $ln\left[sinh(\alpha \sigma )\right]$ − 1/T, the relationship curve is shown in Figure 6. In Figure 6a $ln\dot{\varepsilon }$-$ln\left[sinh(\alpha \sigma )\right]$ the slope of the relationship curve is 10.28; In Figure 6b, $ln\left[sinh(\alpha \sigma )\right]$ − 1/T the slope of the curve (*n*) is 2.99. The molar gas constant *R* is taken as 8.31 J/(mol·K), and based on the calculation, the activation energy for thermal deformation can be obtained Q it is 218.40 kJ/mol.

The hot deformation condition and rheological stress can be represented by the material temperature compensation strain rate factor. By deforming Formula (2), Formula (10) can be obtained. According to the definition of hyperbolic sine function, Formula (11) can be obtained. Strain rate at different deformation temperatures $\dot{\varepsilon }$ thermal deformation activation energy Q substituting into Formula (2) yields different *Z* values, and taking the logarithm of Formula (2) yields Formula (12).

(10) $$\dot{{sinh}^{-1}(\alpha \sigma )={\left(Z/A\right)}^{1/n}=ln}{\left[{\left(\alpha \sigma +{\alpha \sigma }^{2}+1\right)}^{1/2}\right]}^{-1}$$

(11) $$\dot{\sigma =\frac{1}{\alpha }ln\left\{{\left[{\left(Z/A\right)}^{1/n}+{\left(Z/A\right)}^{2/n}+1\right]}^{1/2}\right\}}$$

(12) $$lnZ=lnA+nln\left[sinh(\alpha \sigma )\right]$$

**Figure 7:**
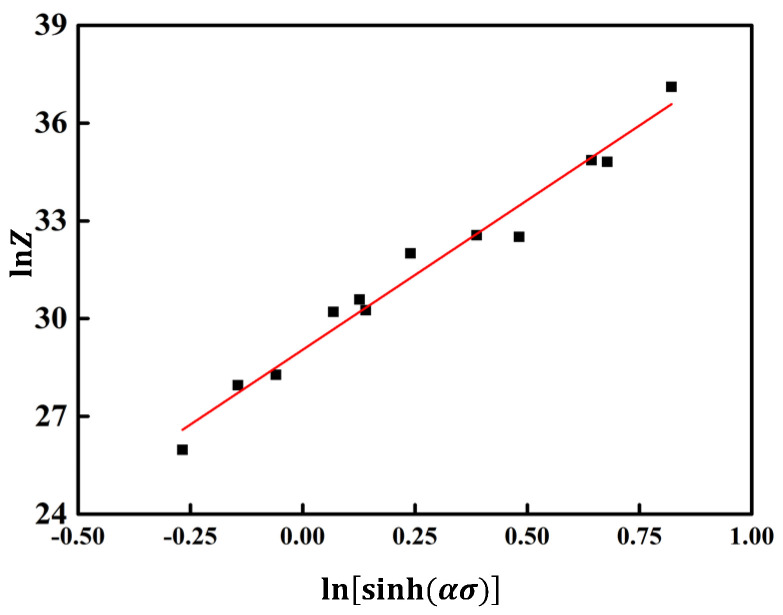
Relationship between strain rate, deformation temperature, and flow stress of aluminum alloy.

Peak stress σ substitute into Formula (12) [30] and use linear regression method to obtain ln*Z*-$ln\left[sinh(\alpha \sigma )\right]$ the relationship curve between strain rate, deformation temperature, and flow stress of aluminum alloy is shown in Figure 7. As shown in the figure, the linear correlation coefficient of the ln*Z* − $ln\left[sinh(\alpha \sigma )\right]$ relationship curve is 0.987. Take ln*Z* − $ln\left[sinh(\alpha \sigma )\right]$ substitute the slope (*n*) of the relationship curve into Formula (12) to obtain the structural factor *A* = 3.813 × 10^12^ s^−1^.

In summary, the obtained structural factor *A*, stress index *n*, and stress level parameters will be adjusted accordingly α and thermal deformation activation energy Q substituting into Formulas (3) and (11), the constitutive Formula of thermal deformation flow stress for aluminum alloy can be obtained, as shown in Formulas (13) and (14).

(13) $$\dot{\varepsilon }=3.813\times 10^{12}{\left[sinh(0.0178\sigma )\right]}^{2.99}exp\left[-218.40\times 10^{3}/(RT)\right]$$

(14) $$\dot{\sigma =56.18ln\left\{{\left[{\left(Z/3.813\times 10^{12}\right)}^{1/2.99}+{\left(Z/3.813\times 10^{12}\right)}^{2/2.99}+1\right]}^{1/2}\right\}}$$

The formula applies to the rheological stress behavior of aluminum alloys with a deformation temperature of 450~550 °C, a strain rate of 0.01~10 s^−1^, and a total deformation of 60%. The relationship between material deformation velocity and deformation temperature is verified, so it is also called temperature-compensated deformation velocity, and many phenomena of plastic machining metals can be explained by this parameter.

### 3.3. Hot Working Diagram

When metal undergoes thermal deformation, when ε and *T* are determined, σ and $\dot{\varepsilon }$ satisfy Formula (15).

(15) $$\sigma =K{\dot{\varepsilon }}^{m}$$

In the formula, *K* is a constant and *m* is the strain rate sensitivity coefficient. Prasad et al. [31] proposed the Dynamic Materials Model (DMM), which suggests that the material during thermoplastic deformation can be regarded as a power dissipator, and the energy flowing into the material from the outside can be divided into two parts: the energy consumed by plastic deformation (*G*) and the energy consumed by tissue deformation (*J*), which can be expressed by Formula (16). *m* can be obtained using Formula (17) [32].

(16) $$P=\sigma \dot{\varepsilon }=G+J=\int_{0}^{\dot{\varepsilon }}\sigma d\dot{\varepsilon }+\int_{0}^{\sigma }\sigma d\dot{\varepsilon }$$

(17) $$m={\left(\frac{\partial J}{\partial G}\right)}_{\varepsilon ,T}={\left(\frac{\partial ln\sigma }{\partial ln\dot{\varepsilon }}\right)}_{\varepsilon ,T}$$

Using cubic spline function for solution, assuming σ, $\dot{\varepsilon }$ the Formula relationship is represented by Formula (18). Using Origin 2019b software to fit the three-term function, obtain $a_{1}$, $a_{2}$, $a_{3}$, $a_{4}$. By substituting it into Formula (19), the strain rate sensitivity coefficient *m* can be obtained.

(18) $$ln\sigma =a_{1}+a_{2}ln\dot{\varepsilon }+a_{3}{\left(ln\dot{\varepsilon }\right)}^{2}+a_{4}{\left(ln\dot{\varepsilon }\right)}^{3}$$

(19) $$m={\left(\frac{\partial ln\sigma }{\partial ln\dot{\varepsilon }}\right)}_{\varepsilon ,T}=a_{2}+{2a}_{3}ln\dot{\varepsilon }+3a_{4}{\left(ln\dot{\varepsilon }\right)}^{2}$$

**Figure 8:**
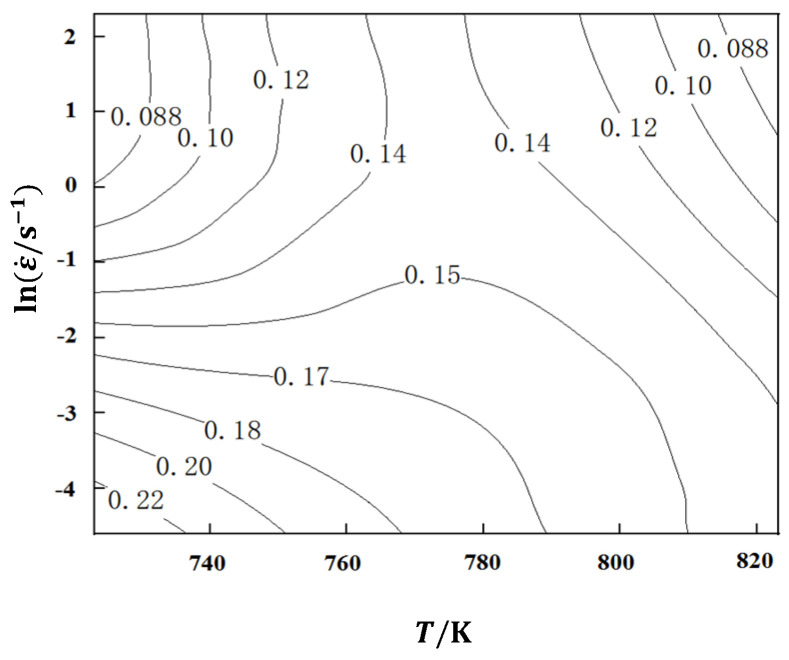
Power dissipation diagram of aluminum alloy.

The energy dissipation during material deformation can be achieved through power dissipation efficiency η represent, η is the ratio of the energy consumed to change the tissue during material deformation to the energy input into the system. The larger the ratio, the more energy is used to change the tissue state of the material during thermal deformation, which is represented by the Formula (20).

(20) $$\eta =\frac{2m}{m+1}$$

stay η-Draw the power dissipation efficiency on the two-dimensional plane composed of $ln\dot{\varepsilon }$-*T* the contour map is shown in Figure 8. Due to the thermal deformation of materials, the power dissipation efficiency of alloys is related to the deformation efficiency of heat input on the microstructure. Therefore, in areas with higher power dissipation efficiency, more energy is used to change the microstructure of the material during hot deformation, resulting in higher energy utilization and more conducive to alloy deformation. 

The power dissipation diagram (Figure 7) can display the microstructure changes of materials under different deformation temperatures and strain rates. However, the power dissipation efficiency cannot display the adverse effects of micro defects, and it cannot accurately determine whether a higher power dissipation rate is due to excellent material processing performance or the generation of micro defects. According to the DMM model theory, the judgment of the machining instability zone proposed by Prasad [33] is expressed by Formula (21). Among them, $\xi (\dot{\varepsilon })$ is the instability factor, which is defined as a dimensionless number, reflecting the meaning: when the entropy production rate of a system is less than the strain rate applied to the system, the plastic flow will be localized, resulting in rheological instability. The machining instability map is a contour map with strain rate and deformation temperature as axes to represent the region where the instability factor value is negative.

(21) $$\xi (\dot{\varepsilon })=\frac{\partial ln(\frac{m}{m+1})}{\partial ln\dot{\varepsilon }}+m<0$$

**Figure 9:**
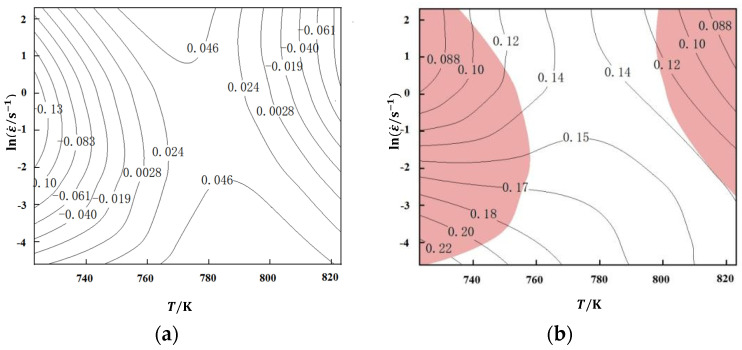
Processing diagram of aluminum alloy: (**a**) Rheological instability diagram; (**b**) Hot working diagram.

Draw the power dissipation efficiency $\xi (\dot{\varepsilon })$ on the two-dimensional plane composed of $ln\dot{\varepsilon }$ − *T* the contour map and rheological instability diagram are shown in Figure 9a. Mark the area of $\xi (\dot{\varepsilon })$ within the planar area formed by $ln\dot{\varepsilon }$-*T*, the region is overlaid with power dissipation diagram and rheological instability diagram, and the thermal processing diagram is shown in Figure 9b.

From Figure 9b, the alloy exhibits two unstable regions during hot compression deformation (highlighted in red in Figure 9b), namely: ① deformation temperature of 450~484 °C and strain rate of 0.01~10 s^−1^; ② the deformation temperature is 522~550 °C, and the strain rate is 0.05~10 s^−1^. The alloy exhibits instability during deformation in this region, and there may be processing defects in the microstructure. Therefore, to avoid material instability during production, aluminum alloys should choose processing parameters within the deformation temperature range of 484~522 °C and strain rate range of 0.01~10 s^−1^ to ensure that the alloy does not experience rheological instability during hot working.

### 3.4. Microstructure of Hot Compressed Specimens

**Figure 10:**
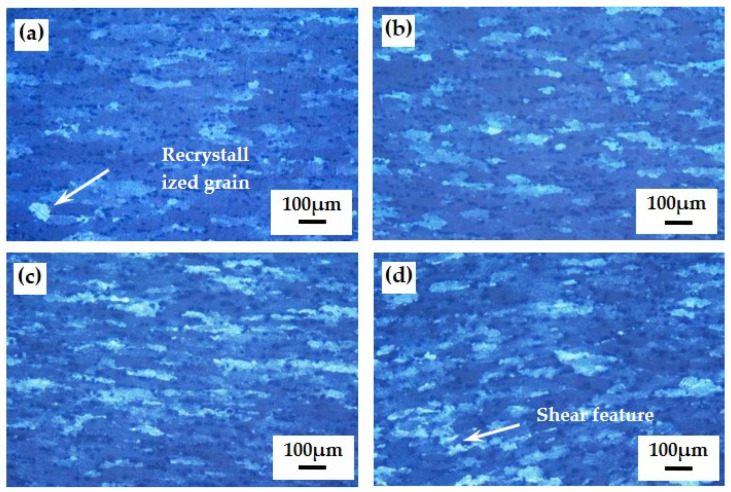
Microscopic grain structure of aluminum alloy at deformation temperature of 500 °C: (**a**) 0.01 s^−1^; (**b**) 0.1 s^−1^; (**c**) 1.0 s^−1^; (**d**) 10 s^−1^.

According to the instability analysis results of hot compression tests, rare earth-containing alloys can ensure stable forming at a deformation temperature of 480~520 °C and a strain rate of 0.01~10 s^−1^. When the deformation temperature is 500 °C, the microstructure of the compressed samples under different strain rates is shown in Figure 10.

When the strain rate is 0.01 s^−1^, the grain structure of the alloy is roughly fibrous, and there are recrystallized grain structures, and the recrystallized grains are spherically shaped. The parallel temperature of the alloy exceeds the recrystallization temperature, the strain rate is low, and the deformation time is longer under the same strain. The recrystallization grains have sufficient time to nucleate and grow, which may lead to low strength of the material and poor resistance to fracture. At the strain rate of 0.1 s^−1^ and 1.0 s^−1^, the grain structure of the alloy is fibrous, and only small recrystallized grains exist in some locations. At the same time, the thickness of the fibrous structure decreases significantly with the increase of the strain rate. At the strain rate of 0.01 s^−1^, the thickness of the fibrous grain is about 30 μm. When the strain rate is 10 s^−1^, the grains change from fibrous shape to irregular shape gradually, the grain boundaries change from straight and continuous to discontinuous, and the grain boundary edges are curved and cut. When the strain rate is large, recovery and recrystallization cannot overcome the work-hardening phenomenon, which usually shows a decrease in plasticity and an increase in deformation resistance. On the other hand, the larger strain rate will lead to the intensification of grain friction, part of the energy consumed in plastic deformation is converted into heat energy, so that the metal temperature rises, and the material exceeds the optimal plastic range and loses the ability to plastic processing. When the metal deformation rate is large, the stress concentration caused by grain boundary slip does not have enough time to transfer, which may cause local instability or cracking of the material. In summary, for the thermoplastic deformation of rare earth aluminum alloy, the selection range of strain rate should be 0.1 s^−1^~1.0 s^−1^.

## 4. Design of Extrusion Process for Aluminum Alloys

### 4.1. Extrusion Process Parameters

The various indicators in the extrusion process parameters, including the heating temperature of the ingot, the temperature of the extrusion cylinder, and the product speed, directly determine the extrusion outlet temperature of the product, which affects the solid solution effect of the profile. The battery pack tray is selected with representative side beam sections and subjected to extrusion tests on a 36 MN single-action forward extruder. The heating method is stepwise heating, which involves isothermal extrusion with a slightly higher temperature at the head end of the casting rod and a slightly lower metal temperature at the tail end. Considering the thermal deformation rheological properties and extrusion formability of the material, and based on the product’s usage conditions and past production experience, adjust the extrusion speed and ingot temperature for extrusion process testing.

An increase in extrusion speed will cause an increase in extrusion force, which is related to the tonnage of the extruder. To ensure that all tests are conducted on the same tonnage extruder, considering the finished product speed and safety, the ingot is preheated in an induction furnace with a heating temperature of 500 °C. The extrusion speeds are selected as 26 mm/s, 42 mm/s, and 58 mm/s (product speed), corresponding to strain rates of 0.03 s^−1^, 0.05 s^−1^, and 0.07 s^−1^, respectively. The other parameters during the extrusion process remain unchanged, with an extrusion ratio of 32.2, a set temperature of 460 °C for the extrusion cylinder, and 450 °C for the mold. The outlet profile is quenched online using strong wind.

### 4.2. Impact of Extrusion Speed on Profile Quality

Strain rate is an important factor in the rheological behavior of metals, corresponding to the speed of the product during the extrusion process. The surface temperature of the profile was measured by a contact thermometer. The quenching temperature and cooling time of profiles under different extrusion speeds are not the same, and the process parameters during the experiment are shown in Table 3. During the extrusion molding process, the ingot needs to be forced through the working strip with a smaller cross-sectional area designed by the mold under the action of extrusion pressure. The force acting on the ingot is partly used for metal flow deformation and partly for friction consumption between the ingot and the mold. Overcoming the friction between the mold and the ingot will generate a certain amount of heat. When the extrusion speed is fast, the temperature generated by the rheology is higher, and the heat generated by the friction between the ingot and the mold cannot diffuse in time. Therefore, the quenching temperature of the profile is relatively high.

**Figure 11:**
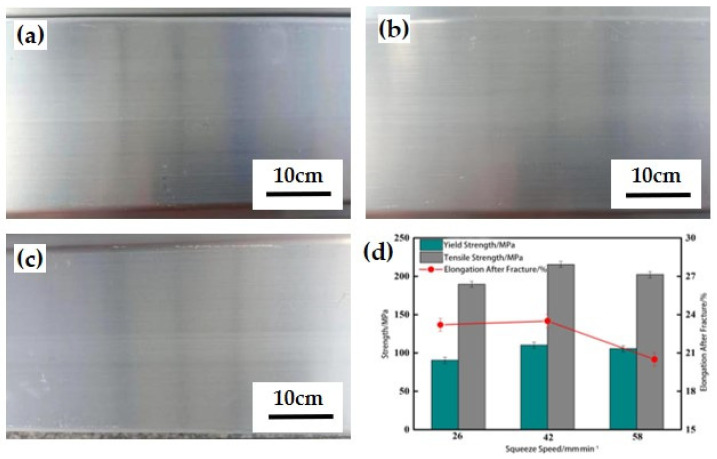
Effect of extrusion speed on profile quality: (**a**) 26 mm/s; (**b**) 42 mm/s; (**c**) 58 mm/s; (**d**) Mechanical properties of profiles under different extrusion speeds.

The influence of extrusion speed on the surface quality of profiles is shown in Figure 11a–c. At all extrusion speeds, no cracking occurred in the profile. When the extrusion speed is 26 mm/s or 42 mm/s, the surface forming quality of the profile is good, and the surface is smooth and flat; The extrusion speed has been increased to 58 mm/s, and there are slight white marks on the surface of the profile. This is due to the fast speed of extruded products, which increases the friction between the ingot and the mold. Under the action of traction, the surface of the profile turns white. The influence of extrusion speed on the mechanical properties of profiles is shown in Figure 11d. When the extrusion speed is 42 mm/s, the comprehensive mechanical properties are optimal, with yield strength, tensile strength, and elongation after fracture of the profile being approximately 115.7 MPa, 218.4 MPa, and 22.8%, respectively.

**Figure 12:**
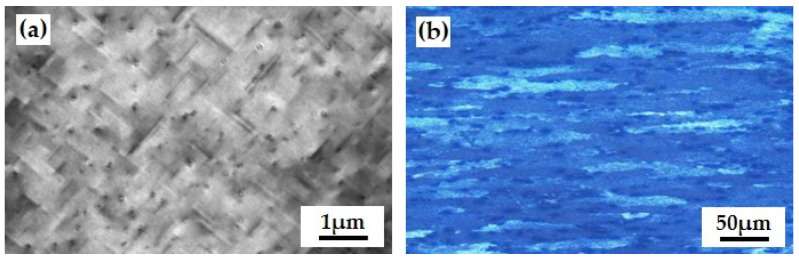
Microscopic morphology of the extruded matrix of aluminum alloy: (**a**) Magnified SEM image of the sample; (**b**) Optical microscopic grain structure.

The main reason for the change in mechanical properties under different product speeds is due to the quenching temperature and quenching time of the profile. When using a lower extrusion speed, the temperature for the solid solution of the profile is low, and the solid solution effect is not sufficient; When using a higher extrusion speed, the online quenching time of profiles is greatly reduced, and the quenching effect is not sufficient. Therefore, to ensure the quality and mechanical properties of the profile forming, the product speed should be selected as 42 mm/s (strain rate of 0.05 s^−1^). Using optimized technical parameters to extrude aluminum alloy, the microstructure morphology of the extruded matrix is shown in Figure 10. Under this system, the microstructure of the grains is fibrous (Figure 12b). From Figure 12a, there are many second phases in the organization, mainly divided into two types, namely the needle-shaped second phase in black and the gray second phase in the petal petal-shaped second phase. The size of the two second phases is small and their distribution is relatively uniform. According to literature records [34,35], the needle-shaped black second phase is the Mg_2_Si phase that re-precipitates during the hot extrusion molding cooling process. The black particle phase is a secondary Al_3_Sc phase [36,37].

**Figure 13:**
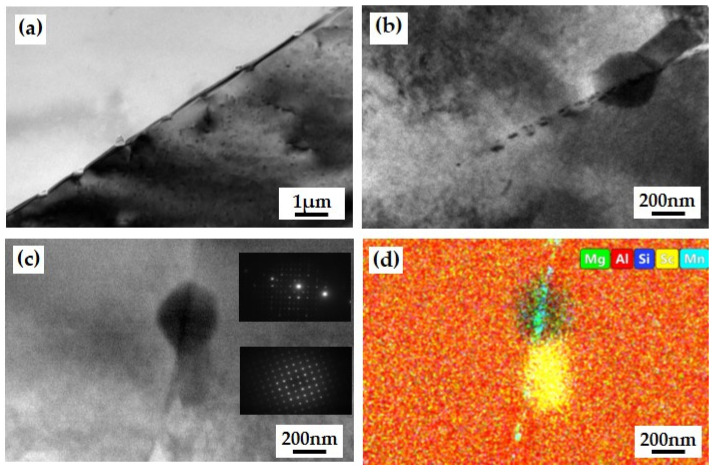
Microscopic morphology of grain boundary position in aluminum alloy in extruded state: (**a**) microstructure; (**b**) Second phase appearance; (**c**) Diffraction pattern ([001]); (**d**) Element distribution.

The microstructure and elemental composition of the extruded grain boundary position of aluminum alloy are shown in Figure 13. From Figure 13a, there are many blocky and granular second phases at the grain boundaries, with small sizes of about 200 nm and relatively uniform distribution at the grain boundaries. The morphology of the second phase at the grain boundary position is shown in Figure 13b. From the figure, in addition to the granular second phase, there are also intermittent black dot-like second phases distributed along the grain boundary. The EDS Map (face scan) results at position Figure 13b are shown in Figure 13c. According to the analysis of the detection results, the second phase at the grain boundary position is mainly composed of three types, namely the A1-Sc-Si phase, Al-Fe-Mn-Si phase, and Mg Si phase. The A1-Sc-Si phase and Al-Fe-Mn-Si phase have smaller sizes, which is because, during the hot extrusion process of the ingot, the large second phase will break into smaller-sized second phases under the action of rheological stress. Mg and Si elements are uniformly distributed at the grain boundaries, with higher content than inside the matrix. It is speculated that Mg_2_Si is re-precipitated during the cooling process of hot extrusion molding.

## 5. Analysis and Discussion

Compared with the traditional Al-Mg-Si alloy, Al-Mg-Si-Mn-Sc alloy has stronger deformation resistance, especially in the process of hot extrusion molding, which requires more extrusion pressure and higher extrusion temperature. To ensure that the alloy does not become instability, the hot extrusion molding process window of the alloy is narrow. Al-Mg-Si-Mn-Sc alloy can still maintain a fibrous structure after thermal deformation, which is related to the recrystallization inhibition of Sc, mainly reflected in the following aspects:

(1) Al_3_Sc particles in the alloy have a certain pinning effect on dislocation and subgrain boundary, which can prevent dislocation recombination and subgrain boundary migration, cause serious entanglement of dislocation in the tissue, hinder the nucleation mode such as polymerization and subgrain growth, and have a strong stabilizing effect, thus delaying the formation of recrystallization nucleus. The presence of the subbasin structure of the alloy will be an important barrier to dislocation movement. At the same time, in the process of recrystallization nucleus growth, small Al_3_Sc particles can inhibit the migration of large grain boundaries and inhibit the growth of the recrystallization core, thus inhibiting the process of recrystallization. Although the size of the Al-Sc-Si phase and Al-Fe-Mn-Si phase in the alloy is larger than that of Al_3_Sc after extrusion, they are also sub-micron and can inhibit recrystallization.
(2) In any alloy system, the binding energy between solute atoms and vacancy mainly depends on two factors, namely the size factor and electron concentration factor. The larger the difference in atomic radius between solute and solvent, the larger the difference in valence electron number, and the larger the binding energy with vacancy. The atomic radius of Sc is much larger than that of Al, and the number of valence electrons between the two is also more different, so the binding energy of Sc and vacancy is inevitably higher, and vacancy pairs are easily formed between the two, with high binding energy. Recrystallization is essentially a process of atomic diffusion. Due to the interaction between Sc and vacancy, the self-diffusion rate of Al atoms is reduced, while the movement of defects is hindered, the formation and growth of subcrystals are delayed, and the recrystallization of alloys is also inhibited.
(3) Sc element inhibits the recrystallization process of aluminum alloy and is also related to raising the recrystallization starting temperature of aluminum alloy [38]. The higher recrystallization starting temperature means that the alloy has better thermal stability, which is related to the coherent relationship between Al_3_Sc and α(Al). Because the coherent interface energy is very low, the interface is in a very stable state, and the rate of aggregation and growth is very slow at high temperatures. Based on the characteristics of Sc in aluminum alloy, it will have a significant impact on the recrystallization process of profiles. Usually, the initial recrystallization temperature can be raised by about 200 °C. Therefore, after extrusion, the high-temperature stage is an obstacle to recrystallization grain nucleation, while the low-temperature stage is an obstacle to recrystallization grain growth.

## 6. Conclusions

(1) Through hot compression simulation experiments and simulation analysis, the extrusion molding process window of aluminum alloys was studied in detail, and the effects of different extrusion speeds and ingot heating temperatures on the extrusion molding quality and mechanical properties of the alloys were verified. The following conclusions were drawn: when the heating temperature is in the range of 450~550 °C and the strain rates of aluminum alloys are 0.01 s^−1^ and 0.1 s^−1^, work hardening and dynamic softening compete, with a certain cycle period, and the true stress-strain curve shows a wavy characteristic; When the strain rate is 1.0 s^−1^, there is still periodic fluctuation in the early stage, but it exhibits steady-state rheological characteristics in the later stage; When the strain rate is 10 s^−1^, it exhibits steady-state rheological characteristics.
(2) According to the calculation, the flow stress constitutive Formula of thermal deformation of Al-Mg-Si-Mn-Sc alloy is determined:
  $$\dot{\varepsilon }=3.813\times 10^{12}{\left[sinh(0.0178\sigma )\right]}^{2.99}exp\left[-218.40\times 10^{3}/(RT)\right]$$
(3) By overlaying the power dissipation diagram and rheological instability diagram of aluminum alloy, there are two instability regions in the alloy during hot deformation, namely: ① deformation temperature 450~484 °C, strain rate 0.01~10 s^−1^; ② the deformation temperature is 522–550 °C, and the strain rate is 0.05–10 s^−1^. Based on the analysis of hot compression microstructure, the hot deformation process window of aluminum alloy is deformation temperature 484~522 °C, strain rate 0.01~0.1 s^−1^.
(4) By verifying the extrusion process, the optimal extrusion molding process for aluminum alloy was determined to be: a deformation temperature of 490–510 °C, a strain rate of 0.05 s^−1^. Under this system, the surface forming quality and performance of the profiles are good, and the microstructure of the grains is fibrous.
(5) There are three main types of second phases at grain boundaries, namely A1-Sc-Si phase, Al-Fe-Mn-Si phase, and Mg-Si phase.
(6) The main effect of rare earth Sc on the alloy is to inhibit the recrystallization process of the alloy so that the alloy can still retain the fiber grain structure after thermal deformation. Aiming at this property, the hot extrusion molding process window of the alloy is determined by the thermal deformation flow stress Formula, superimposed power dissipation diagram, and rheological instability diagram. The parameters obtained in this experiment consider the extrusion formability and internal microstructure of the alloy, and good results are obtained.

## Figures and Tables

**Table 1:** Chemical Composition of Aluminum Alloy (wt.%).

Element	Si	Mg	Fe	Cu	Mn	Cr	Ti	Sc	Al
content	0.73	0.65	0.09	0.15	0.26	0.05	0.02	0.29	Bal

**Table 2:** Hot compression specimen test technology.

Number		Strain Rate/s^−1^			
		0.01	0.1	1.0	10
test temperature/°C	450	1	4	7	10
	500	2	5	8	11
	550	3	6	9	12

**Table 3:** Extrusion process parameters.

Number	Extruded Speed/mm s^−1^	Profile Quenching Temperature/°C		
		Head-End	Midst	Tail
1	26	505	508	510
2	42	512	518	520
3	58	515	520	525

## Data Availability

The original contributions presented in the study are included in the article, further inquiries can be directed to the corresponding author.
